# The Gillies's Approach to posttraumatic reconstruction of ballistic injuries in evidence a century later

**Published:** 2018-05-15

**Authors:** Michele A. Manahan, Stephen M. Milner

**Affiliations:** Johns Hopkins University School of Medicine

**Figure:**
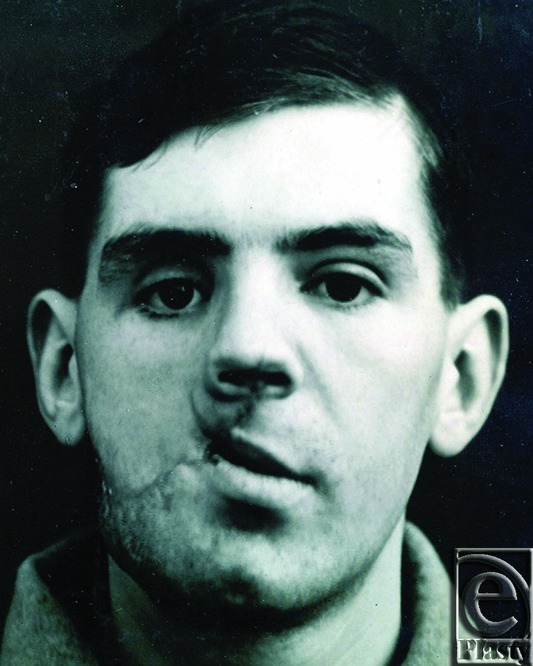
The Gillies' approach to posttraumatic reconstruction in evidence a century later A comparison is made between preoperative evaluation, reconstruction, and outcome in the era of Sir Harold Gillies, one hundred years ago, and those of today.

Forged from the fires of World War I, Gillies’ methods of posttraumatic reconstruction were revolutionary and represent a concrete foundation upon which is built the field of modern reconstructive surgery. Similarities and differences in preoperative evaluation, intraoperative methodology, and outcomes between Gillies's era and modern times may be appreciated through the juxtaposition of Gillies's original notes and photographs with those from a similar case recently treated at a tertiary care, inner city, teaching hospital.

The demographics of victims are similar, involving predominantly young healthy men, with injuries often limited to the head and upper torso. Multi-disciplinary treatment was established by Gillies and remains a constant aspect of care today. Another distinction is that Gillies most frequently encountered patients who had been stabilized elsewhere and who demonstrated significant unmanaged healing with subsequent scarring and contracture. This required delayed reconstructions of both the traumatic defect and nature's response to it.

Figures 1 and 2 illustrate the appearance of a 23-year-old soldier who presented to Gillies in August of 1918, several months after sustaining a gunshot wound in France. In the words of Gillies, “There is a large wound of soft tissues extending from the right angle of the mandible upwards and inwards to the angle of the mouth and involving the upper lip which is much lacerated.”^1^ As seen in the photographs, this wound has entered a chronic phase in which scarring and contractures are evident.

Patients nowadays are evaluated almost immediately by craniofacial trauma surgeons, and stabilized with concern for tissue preservation and reconstruction. Recently, an 18-year-old male sustained a gunshot wound to the face. Figures 2A and 2B demonstrate the immediate appearance of the wound upon initial presentation to a modern trauma facility.

This patient also sustained drastic soft tissue injuries including full thickness loss of the lower lip, including the mucosa, a complex tongue laceration, and damage to the right cheek. However, wounds were obviously much fresher, and healing (which alters reconstructive needs) had not occurred.

Gillies identified in his patient a bony defect which had previously been stabilized and was healing, albeit inadequately, by the time of presentation (Figure 3). He described an “X-ray demonstrates a transverse fracture of the right body of the mandible in the molar region with much destruction of the ascending ramus, a thin bridge of which remains, the coronoid process having been carried away.” A significant portion of his right maxilla was also found to be missing.

The recent patient demonstrated severe comminution and complete destruction of the symphysis and right parasymphyseal region of the mandible as well as a partial right maxillary defect, which are demonstrated by three-dimensional computerized tomography (Figure 4).

Current imaging modalities demonstrate the extreme advances in technology. Plain radiographs, however, are still frequently used as adjuncts to clinical examinations.

Gillies began his work with “extraction of teeth and drainage of [the] mandible” followed by use of a “double gunning metal splint with extra buccal wires and elastic traction to [a] head cap”^1^ to address the fibrous nonunion which was preventing appropriate healing of the mandibular fractures.

Our patient underwent initial open reduction and internal fixation of the mandible with a spanning reconstruction plate and debridement of soft tissues with reapproximation of the remaining edges.

The soft tissue disruptions proved more difficult to overcome in both situations. Gillies identified four major injury components requiring treatment which he addressed individually in separate procedures temporally remote from each other. He began by addressing the contracture of the upper lip which was stuck to the maxilla. He described, “Division of the dense fibrous band uniting [the] lip to [the] alveolus of [the] upper jaw” accompanied by insertion of “an epithelial inlay with the aid of a splint attachment to form a new sulcus and to release the lip. In order to give sufficient depth to the lip and to depress the angle of the mouth, a pedicle flap was cut transversely across the mental region and swung upwards to the lip.”^1^

Months later, Gillies returned the patient to the operating room to recreate a vermilion border for the released, repositioned, and augmented upper lip. Gillies stated, “A flap of mucous membrane was turned up from the inner aspect of the lower lip to supply the vermilion border for the upper lip.” Again, the patient was allowed to heal from this procedure before treating his microstomia. Gillies noted, “[A] portion of [the] former flap [was] inserted under [the] margin of [the] vermilion border of [the] lower lip.” In another stage, Gillies created an “inlay under lip and cheek for [a] prosthesis to restore prominence of [the] right upper alveolar region.”^1^

The contemporary patient encountered similar obstacles following his initial surgery in which primary closure with remaining tissue was attempted. He too required scar release of the lip, additional tissue transfer to augment insufficient tissue of the lip and oral commissure, and vermilion restoration. However, he received treatment of these disparate issues in a single stage operation with multiple components. Additionally, vermilion reconstruction was performed with a local mucosal flap as Gillies described. A major advance, in the era of microsurgery, was free tissue transfer of the fibula to the mandible with a skin paddle to augment the lip and chin and decrease microstomia.

Gillies's patient concluded therapy in 1922, four years after sustaining his injury. Prior to completing his treatment course, he underwent multiple small procedures for creation of an upper prosthesis and partial lower denture as well as fine tuning to skin grafts, tissue redundancies, and scars, followed by scar massage – all procedures commonly recommended to augment postoperative results today. The modern eighteen-year-old declined further surgery for contouring and scar revision. Figures 5 and 6 demonstrate the results obtained.

Despite the passage of nearly a century and drastically shifting mechanisms of injury, patient presentations remain quite constant. In many instances, current operative techniques for reconstruction of traumatic defects may be intimately associated with Gillies's approaches. Postoperative final results are also strikingly similar, despite technical developments.

## History of Gillies

Sir Harold Gillies was born in Dunedin, New Zealand, on June 17, 1882.^2^ He studied medicine at Cambridge and attained formal surgical training at St. Bartholomews Hospital where he was appointed house surgeon to Douglas Harmer, head of the newly created Ear, Nose, and Throat Department.^2^^,^^3^ At the beginning of World War 1, at the age of 32. he joined the Royal Army Medical Corps,

Initially posted to Belgium, he worked under Auguste Valadier and Hippolyte Morestin in France who specialized in extirpative surgery for cancer of the head and neck.^3^

Upon his transfer back to England in 1916, he established the first facial injury ward at the Cambridge Military Hospital, Aldershot. When facilities were overwhelmed, the Queen's Hospital, devoted entirely to plastic surgery, was opened at Sidcup. Treating over 5,000 men with more than 11,000 operations from 1916 until 1925, Gillies developed many of the groundbreaking plastic surgery techniques, involving local flaps, bone grafts, inlay and onlay skin grafting and tube pedicles.^3^^,^^4^^,^^5^

Years later he became immersed in the problems of the Second World War, as consultant in charge of Rooksdown Centre for Plastic Surgery.

## Figures and Tables

**Figure 1A and B:**
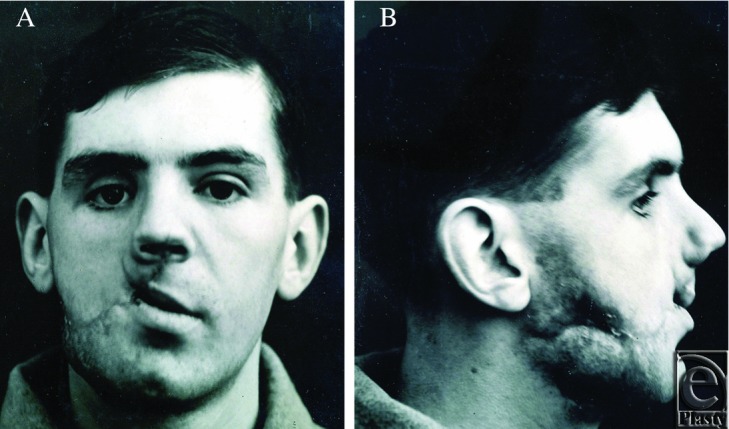
23-year-old male presenting to Gillies in 1918 following a gunshot wound to the face, sustained in trench warfare. Initial presentation

**Figure 2A:**
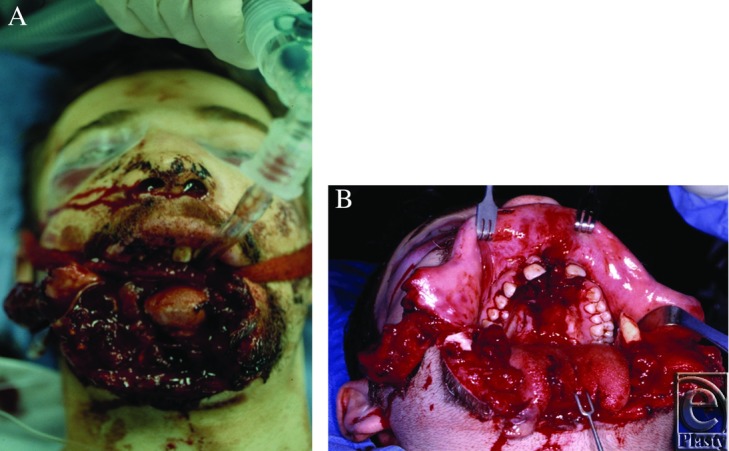
18-year-old male presenting recently to a tertiary care facility following a self-inflicted gunshot wound. **2B.** Same patient within hours of presentation following irrigation and cleansing of the wounds.

**Figure 3:**
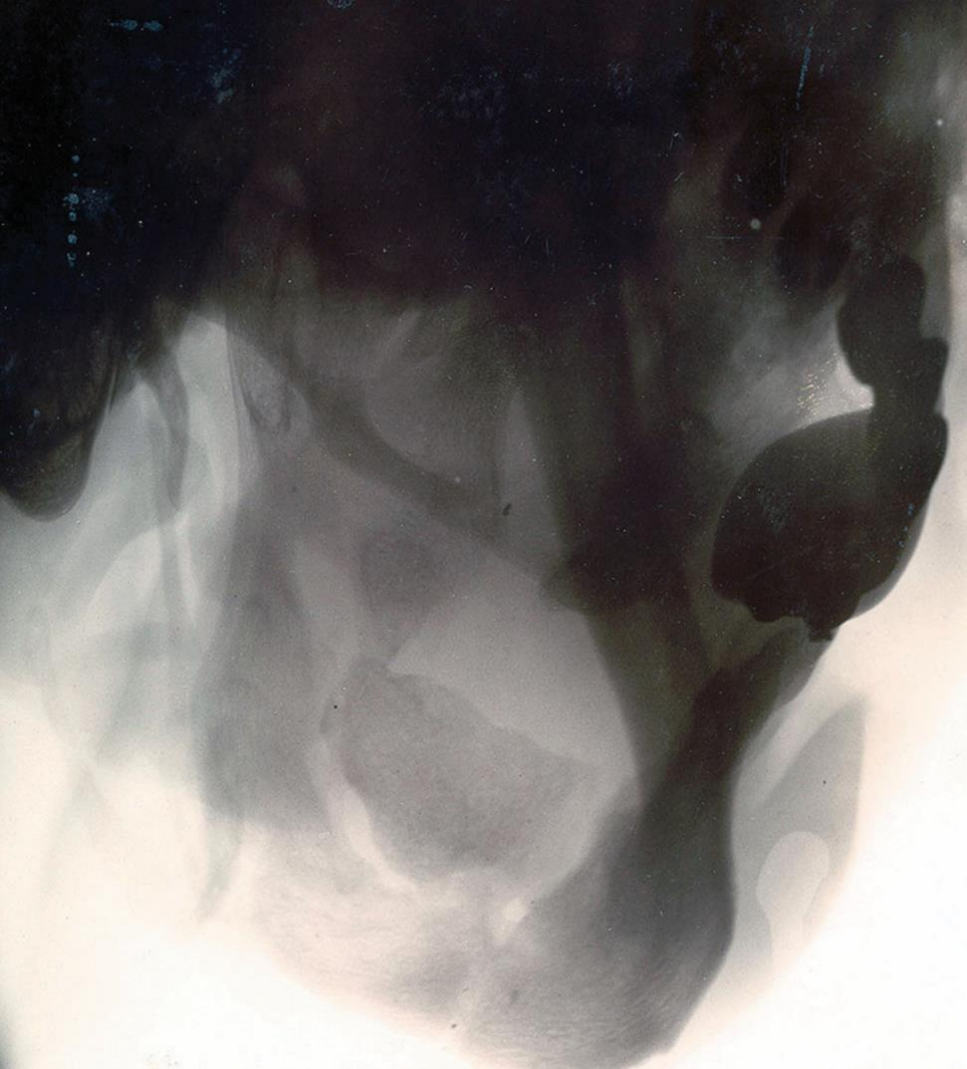
Plain radiograph demonstrating the bony injuries sustained by Gillies’ 23-year-old soldier.

**Figure 4:**
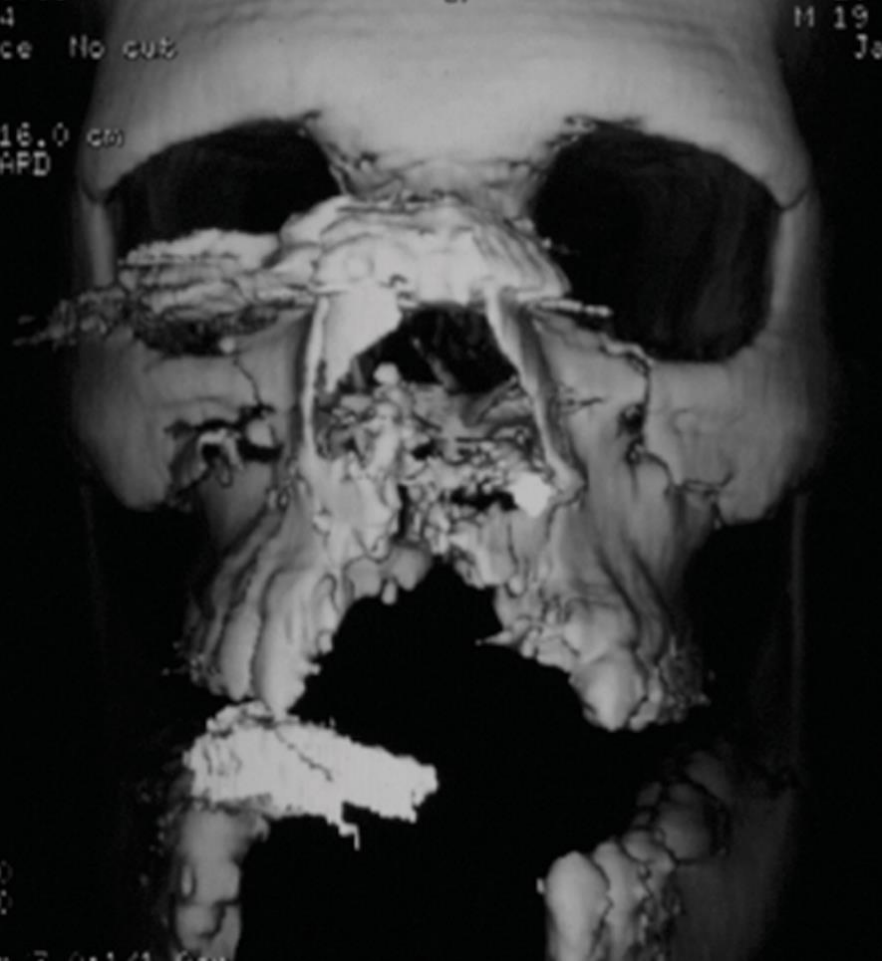
Three dimensional CT scan demonstrating extensive bone

**Figures 5A and B:**
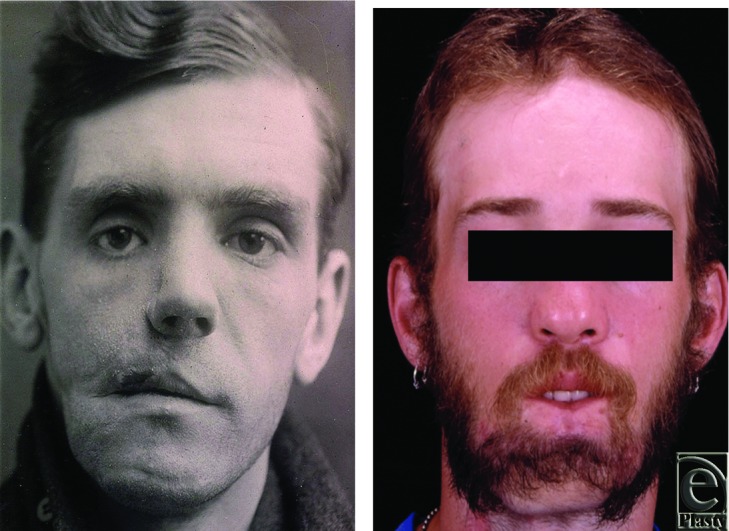
Final appearances
